# Photothermal Radiometry Data Analysis by Using Machine Learning

**DOI:** 10.3390/s24103015

**Published:** 2024-05-09

**Authors:** Perry Xiao, Daqing Chen

**Affiliations:** School of Engineering, London South Bank University, London SE1 0AA, UK; chend@lsbu.ac.uk

**Keywords:** photothermal techniques, skin hydration, machine learning, deep learning, regression, classification

## Abstract

Photothermal techniques are infrared remote sensing techniques that have been used for biomedical applications, as well as industrial non-destructive testing (NDT). Machine learning is a branch of artificial intelligence, which includes a set of algorithms for learning from past data and analyzing new data, without being explicitly programmed to do so. In this paper, we first review the latest development of machine learning and its applications in photothermal techniques. Next, we present our latest work on machine learning for data analysis in opto-thermal transient emission radiometry (OTTER), which is a type of photothermal technique that has been extensively used in skin hydration, skin hydration depth profiles, skin pigments, as well as topically applied substances and skin penetration measurements. We have investigated different algorithms, such as random forest regression, gradient boosting regression, support vector machine (SVM) regression, and partial least squares regression, as well as deep learning neural network regression. We first introduce the theoretical background, then illustrate its applications with experimental results.

## 1. Introduction

Photothermal techniques [1] are infrared remote sensing techniques that have been used for biomedical applications, as well as industrial non-destructive testing (NDT). They can be dated back to the 1970s [2,3]. Photothermal techniques have since developed into different approaches, such as photothermal radiometry [4,5,6,7], photothermal tomography [8], photothermal imaging [9], photothermal radars [10], photothermal lenses [11,12], photothermal cytometry [13], and so on. The main advantages of photothermal techniques lie in their non-invasive, remote-sensing, and most importantly, spectroscopic nature, which make photothermal techniques a potentially powerful tool in many industrial, agricultural, environmental, and biomedical applications. Pawlak has highlighted the advantages of spectrally resolved photothermal radiometry measurements on semiconductor samples [14].

Machine learning [15,16] is a branch of artificial intelligence, which includes a set of algorithms for learning from the past data and analyzing the new data without being explicitly programmed to do so. Machine learning can be generally divided into supervised learning, unsupervised learning, semi-supervised learning and reinforcement learning. Machine learning has also been used in photothermal techniques recently. Verdel et al. have developed a predictive model for the quantitative analysis of human skin using photothermal radiometry and diffuse reflectance spectroscopy [17,18], as well as a hybrid technique for characterization of human skin by combining machine learning and an inverse Monte Carlo approach [19], and made their machine learning model publically available through the GitHub platform [20]. Ahmadi et al. have developed a customized deep unfolding neural network, called Photothermal-SR-Net, for enabling super resolution (SR) imaging in photothermal radiometry [21]. Their model was based on an original deep unfolding neural network (USRNet) [22]. Jawa et al. have used machine learning and statistical methods for studying voids and photothermal effects of a semiconductor rotational medium with thermal relaxation time [23]. Kovács et al. [24] have investigated deep learning approaches, based on U-net [25], for recovering initial temperature profiles from thermographic images in non-destructive material testing. There are also several studies using deep learning neural networks on infrared thermal images for machine health monitoring [26,27], as well as for pavement defect detection and pavement condition classification [28]. Qu et al. have developed low-cost thermal imaging with machine learning for non-invasive diagnosis and therapeutic monitoring of pneumonia [29]. Gajjela et al. have leveraged mid-infrared spectroscopic imaging and deep learning for tissue subtype classification in ovarian cancer [30]. Li Voti et al. have developed photothermal depth profiling by genetic algorithms [31]. Xiao et al. have conducted a review of the field including photothermal depth profiling techniques [32,33].

In this paper, we use machine learning to analyze our own measurement data by using opto-thermal transient emission radiometry (OTTER), which is a type of photothermal radiometry technique that has been used in skin hydration, hydration depth profiling, skin pigments, and trans-dermal drug delivery studies [32,33,34,35,36,37,38,39]. Compared with other technologies, OTTER has the advantages of being non-contact, non-destructive, quick to make a measurement (a few seconds), and being spectroscopic in nature. It is also color blind and can work on any arbitrary sample surfaces. It has a unique depth profiling capability on a sample surface (typically the top 20 µm) [33], which makes it particularly suitable for skin measurements. OTTER is information rich, however, to analyze the signal and get the information is often difficult. To solve this problem, we proposed using machine learning for data analysis. Comparing conventional mathematical analysis, the main advantage of machine learning is that it can study and learn to analyze the data automatically, without the need to build complex mathematical models. We have investigated different algorithms such as random forest regression, gradient boosting regression, support vector machine (SVM) regression, partial least squares regression, as well as deep learning neural networks regression. We first introduce the theoretical background, then illustrate its applications with experimental results.

## 2. Materials and Methods

This section describes the OTTER apparatus used, the machine learning algorithms developed, the volunteer information, and the measurement procedures.

### 2.1. OTTER Apparatus

**Figure 1:**
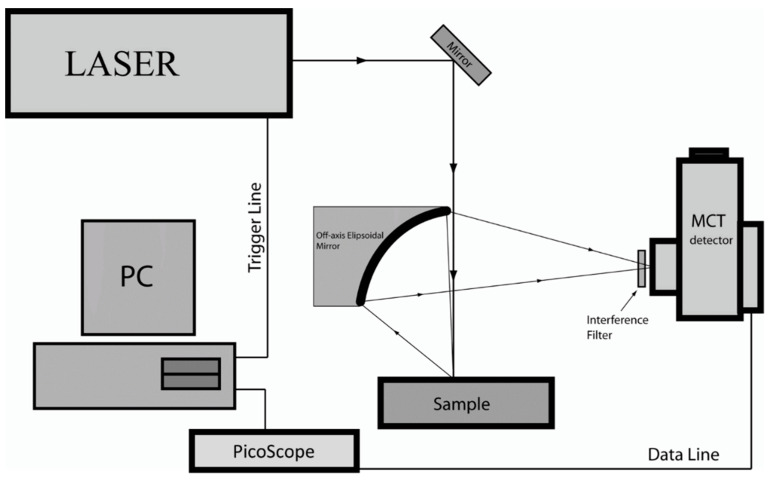
The schematic diagram of OTTER measurements [33].

Figure 1 shows the schematic diagram of opto-thermal transient emission radiometry (OTTER). It uses a pulsed laser (Er:YAG laser, 2.94 µm, a few milli joules per pulse) as a heat source to heat the sample, an ellipsoidal mirror, and a fast infrared MCT (mercury cadmium telluride (InfraRed Associates, Inc., Stuart, FL, USA) detector to measure the consequent blackbody radiation increase of the sample [31,32]. The MCT detector used is the most sensitive infrared detector on the market. It is liquid nitrogen cooled and has a wide sensitivity spectrum range (3–15 μm), high bandwidth (10 MHz), and a purposely designed amplifier. A narrow band interference filter is also used in front of the MCT detector to select different detection wavelengths. By analyzing the OTTER signals, we can get the optical properties, thermal properties, and layered structure information from the sample. The selection of detection wavelength is achieved by using narrow bandpass mid-infrared interference filters. By selecting different detection wavelengths using different narrow band interference filters, we can measure different properties of the sample, for example, the water concentration information in skin (13.1 µm), or solvent concentration information within skin (9.5 µm). The OTTER detection depth is about 20 µm. No other techniques can perform depth-profiling in this range on in-vivo samples [32]. The OTTER skin measurements, therefore, should only be confined within the stratum corneum, which is the outermost skin layer.

For most OTTER measurements, it can be simplified as a one-dimensional semi-infinite problem [31]. For a semi-infinite, optically homogenous material, the OTTER signal can be generally expressed as [5,6,7],

(1) $$S\left(t\right)=Ae^{t/\tau }erfc\sqrt{t/\tau }$$

where A is the amplitude of the signal, τ = 1/(β^2^ D) is the signal decay lifetime, β is the sample’s emission absorption coefficient, and D is the sample’s thermal diffusivity. By fitting the OTTER signal using Equation (1), we can get the best fit β, and from β we can get the water content H in the sample, i.e., of skin, hair, or nail [32].

(2) $$H=\frac{\beta_{w}-\beta }{\beta_{w}-\beta_{d}}$$

where β_w_ is the emission absorption coefficient of water, β_d_ is the emission absorption coefficient of the dry sample. By using segmented least square (SLS) fitting, we can also get the water content at different depths, details are available elsewhere [33,34,35].

For a semi-infinite, optically non-homogenous material, the first assumption is that β is a linear function of depth [32],

(3) $$\beta (z)=\beta_{0}+w_{\beta }z$$

where β_0_ is the absorption coefficient of the surface of the skin, and $w_{\beta }$ is the gradient of the absorption coefficient, then the corresponding OTTER signal can be calculated as:

(4) $$S\left(t\right)=A\left(\frac{2W\sqrt{t\tau }}{\sqrt{\pi }(2Wt+1)}+\frac{1}{\sqrt{2Wt+1}}e^{\frac{t/\tau }{2t/\tau +1}}erfc\left(\frac{\sqrt{t/\tau }}{\sqrt{2Wt+1}}\right)\right)$$

where $W=w_{\beta }D$ is the effective gradient, and τ = 1/(β^2^ D) is the signal decay lifetime. By fitting the OTTER signal with Equation (4), we can get the skin surface absorption coefficient β_0_ and the effective gradient W.

For most complex materials, where β is not a linear function of depth, we can use the enhanced segmented least squares (SLS) fitting algorithm [33], to get the skin hydration depth profiles in the following steps:

1. Load the OTTER signal;
2. Find the starting point and end point of the signal;
3. Fit the entire signal with Equation (1) to get an average sample’s emission absorption coefficient β;
4. Divide signal into 10 slices;
5. Fit the first slice of the signal with Equation (1) to get the first β, then calculate the corresponding detection depth z;
6. Fit the first and the second slice of the signal with Equation (1) to get the second β, then calculate the corresponding detection depth z;
7. Repeat step 6 until all the slices are used.

**Figure 2:**
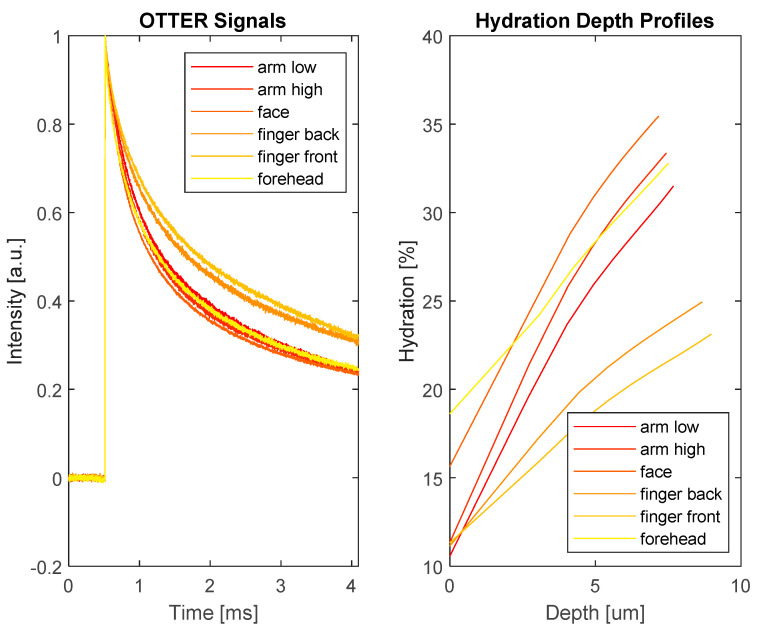
The typical OTTER measurement signals (**left**) and the corresponding hydration depth profiles (**right**), analyzed by using enhanced segmented least squares (SLS) fitting algorithm, of skin sites at arm low, arm high, face, finger back, finger front, and forehead.

With the above algorithm, we can then plot β against depth z to get a depth resolved emission absorption coefficient. With Equation (2), we can also interpret the plot as skin hydration levels at different depths (in micron meters), as shown in Figure 2.

**Figure 3:**
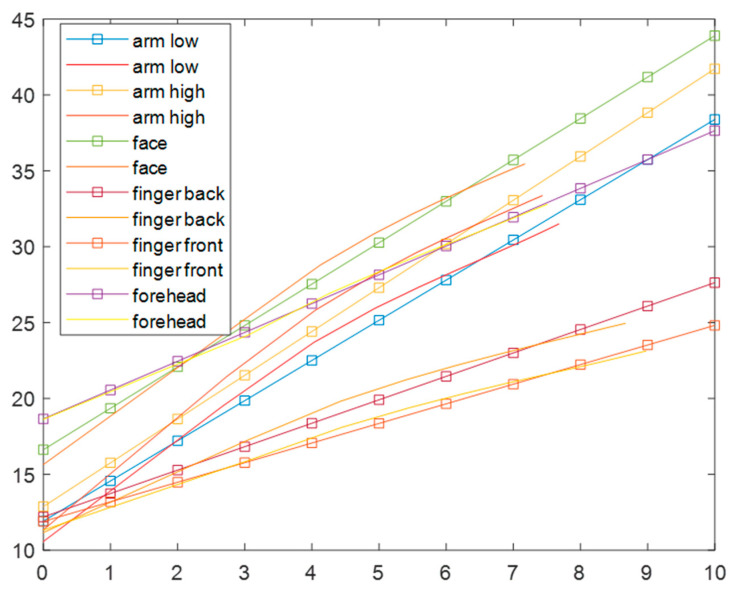
The simplified linear skin hydration distribution by fitting the skin hydration profiles in Figure 2 with Equation (3). The smooth curves are original profiles, the curves with squared markers are fitted straight line profiles.

As the we can see, the skin water hydration level depth profiles are not linear; to simplify the problem, we fit the skin hydration depth profile results in Figure 2 with Equation (3), to get the simplified linear distribution of skin water content, as shown in Figure 3.

### 2.2. Machine Learning Algorithms

The history of artificial intelligence (AI) development [39] can be roughly divided into three stages, artificial neural networks (1950s–1970s), machine learning (1980s–2010s) and deep learning (2010s–present). Generally speaking, machine learning is considered as a subset of AI, and deep learning is considered as a subset of machine learning. Machine learning was originally developed in 1980s and consists of a set of mathematical algorithms that can automatically analyze data without being specifically programmed to do so. Machine learning can be divided into supervised learning, unsupervised learning, semi-supervised learning, and reinforcement learning [40]. In this paper, we will mainly focus on supervised learning, for the purpose of regression and classification. For regression, we have investigated different algorithms such as lasso (least absolute shrinkage and selection operator) [41], elastic net [42], decision tree [43], support vector machine [44], gradient boosting [45], linear regression [46], random forest [47], k-nearest neighbors [48], extreme gradient boosting [49], partial least squares (PLS) regression [50], voting regression [51], and ridge regression with built-in cross-validation (RidgeCV) [52], as well as deep learning neural networks [53,54], to analyze the OTTER data. For classification, we have investigated different supervised learning algorithms for classifying OTTER data.

Lasso regression and ridge regression can be viewed as improved versions of linear regression [55]. For linear regression, the cost function RSS (residual sum of squares) can be written as:

(5) $${RSS\;}_{Linear}\left(W\right)=\sum_{i=1}^{N}{\left(y_{i}-\hat{y}\right)}^{2}=\;\sum_{i=1}^{N}{(y_{i}-\sum_{j=1}^{M}\left(w_{j}x_{ij})\right)}^{2}$$

where $y_{i}$ is the individual y values, N is total number of y values, $w_{j}$ is the corresponding weight for the $x_{ij}$, M is the total number of x values. In order to minimize this cost, we generally use an algorithm called “gradient descent” [56]. Gradient descent means to calculate the partial differentiation of the above equation against weight $w_{j}$, and adjust weight in each iteration until it reaches the optimum stage. However, when the gradient is close to zero, the gradient descent algorithm will stop working. This is commonly known as vanishing gradient [57].

Ridge regression calculates the cost function RSS as the following, with the sum of weight squares:

(6) $${RSS}_{Ridge}\;\left(W\right)=\sum_{i=1}^{N}{\left(y_{i}-\hat{y}\right)}^{2}=\;\sum_{i=1}^{N}{{(y}_{i}-\sum_{j=1}^{M}\left(w_{j}x_{ij})\right)}^{2}{+\lambda \sum_{j=1}^{M}\left(w_{j}\right)}^{2}$$

The λ is the calculation parameter. When we do the partial differentiation of the above equation, it is equivalent to reduce the effect of weight, and can help in the event vanishing gradient problem.

Lasso regression calculates the cost function RSS as the following, with the sum’s absolute value of the magnitude of weights:

(7) $${RSS}_{Lasso}\;\left(W\right)=\sum_{i=1}^{N}{\left(y_{i}-\hat{y}\right)}^{2}=\;\sum_{i=1}^{N}{{(y}_{i}-\sum_{j=1}^{M}\left(w_{j}x_{ij})\right)}^{2}{+\lambda \sum_{j=1}^{M}\left\lfloor w_{j}\right\rfloor }^{2}$$

Ridge regression includes all (or none) of the features in the model, hence has the advantage of coefficient shrinkage and reducing model complexity.

Lasso regression also has several benefits, apart from shrinking coefficients, it also performs feature selection. This is equivalent to exclude certain features from the model.

Elastic net regression uses the linear combination of the penalty functions of ridge regression and lasso regression. By using this approach elastic net can help on overfitting and underfitting problems.

Decision tree and random forest are very popular machine learning algorithms. They are commonly used for classification. For regression, the tree’s predicted outcome can be considered a real number, and it can contain different levels of depth; not enough layers of depth can result in underfit, and too many layers of depth can lead to overfit.

Support vector machine (SVM) is another popular machine learning algorithm, that is commonly used in classification. For regression, support vector regression’s (SVR) goal is to find a function that approximates the relationship between the input variables and an output variable, with minimum error. SVR can handle non-linear relationships between the input variables and the target variable, making it a powerful tool for analyzing complex problems.

Gradient boosting is a relatively new machine learning algorithm that is particularly suitable for tabular datasets. Gradient boosting is a type of ensemble method where you create multiple weak models in order to get better performance as a whole. It can find any non-linear relationship between your model target and features, and has great usability. It can also effectively deal with missing values, outliers, and high cardinality categorical values on your features. There are different versions of gradient boosting trees such as XGBoost or LightGBM.

Partial least squares regression (PLS regression) is a popular regression technique that is commonly used in spectral data analysis. It first projects the input data into a new space, then tries to fit the data by using a linear regression model in the new space. It is a quick, efficient, and optimal regression technique. PLS regression is recommended in cases of regression where the number of explanatory variables is high, and there is likely multicollinearity among the variables [58,59].

Voting regressions [60] belong to the family of ensemble learning [61], which combines the predictions from multiple individual regression models to improve the performance. Voting regressors can use simple averaging or weighted averaging to decide the final outcome.

### 2.3. Measurement Procedure

All the measurements were performed on healthy volunteers (male and female, age 25–55), under normal ambient laboratory conditions of 20–21 °C and 40–50% RH. The volunteer was instructed avoid excess water intake and measurements were taken in the morning. The volar forearm skin sites used were initially wiped clean with ETOH/H_2_O (95/5) solution. The volunteer was then acclimatized in the laboratory for 20 min prior to the experiments.

## 3. Results and Discussions

### 3.1. Regression—Homogenous Model

**Figure 4:**
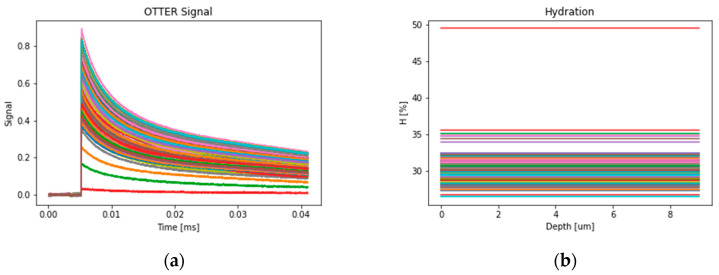
The OTTER skin measurement signals (**a**) and corresponding skin hydration levels in percentages (**b**). The different colors represent different measurements.

All the OTTER measurements are done and analyzed using the steps described in Section 2.1. OTTER signals are analyzed by using Equation (1) and skin hydration is calculated using Equation (2). Figure 4 shows 97 OTTER skin measurement signals and the corresponding skin hydration levels in percentages, calculated using Equation (1) and Equation (2). These OTTER signals were measured from the volar forearm of healthy volunteers, 20–30 years old, under the standard laboratory condition (21 °C, 40%).

**Figure 5:**
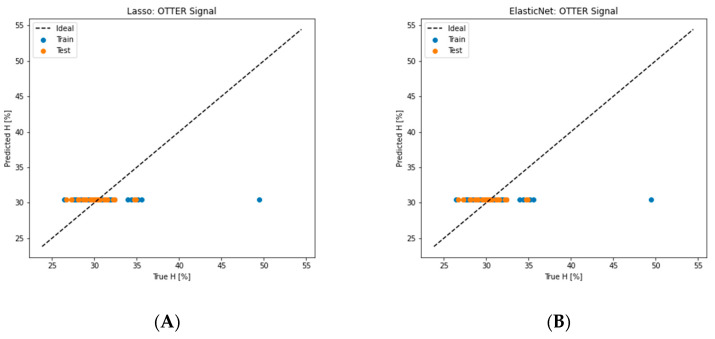

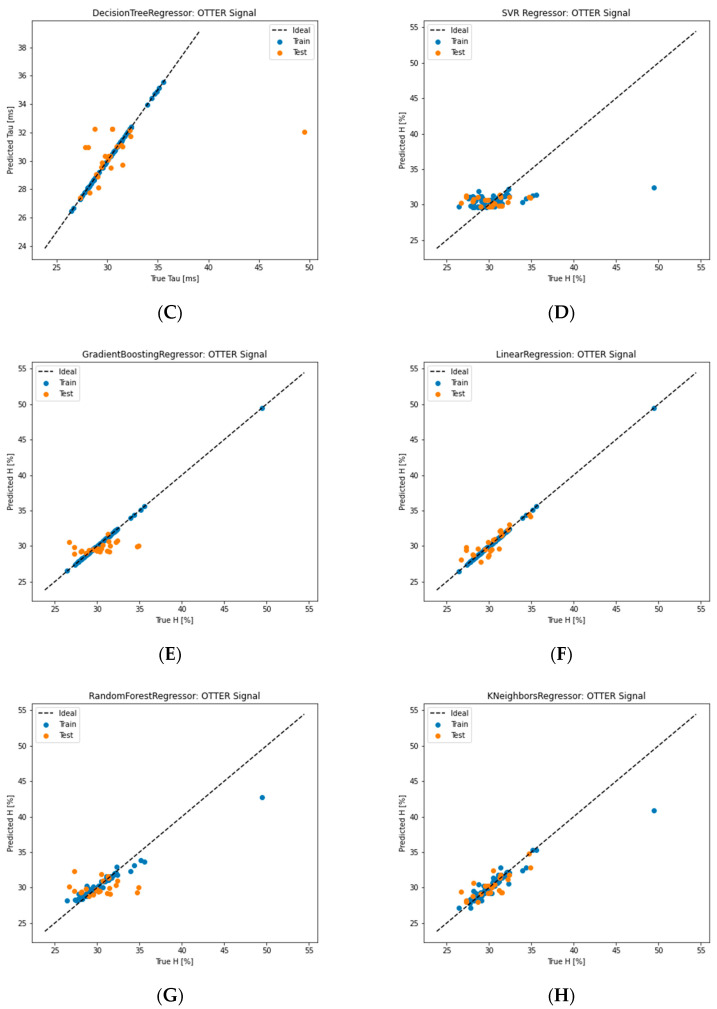

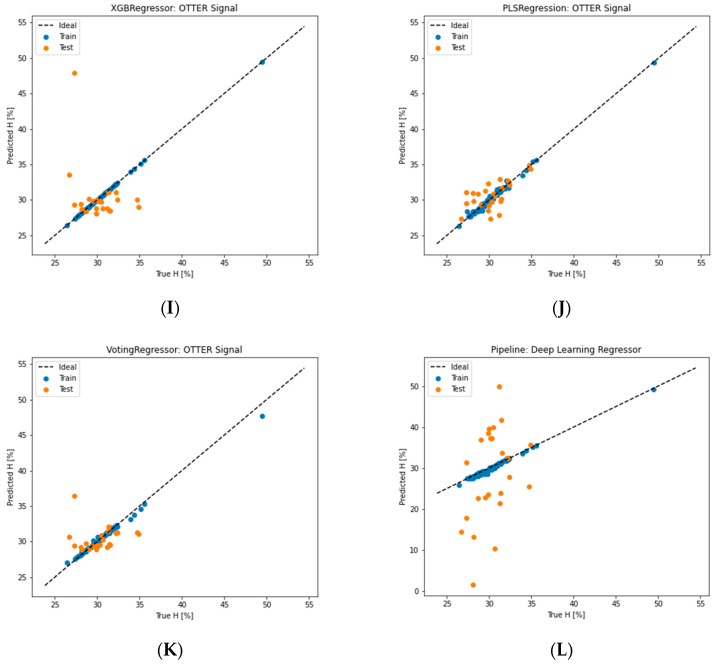
The regression results of different machine learning algorithms models. (**A**) Lasso, (**B**) elastic net, (**C**) decision tree, (**D**) support vector machine, (**E**) gradient boosting, (**F**) linear regression, (**G**) random forest, (**H**) k-nearest neighbours, (**I**) extreme gradient boosting, (**J**) partial least squares (PLS) regression, (**K**) voting regression, (**L**) deep learning.

**Figure 6:**
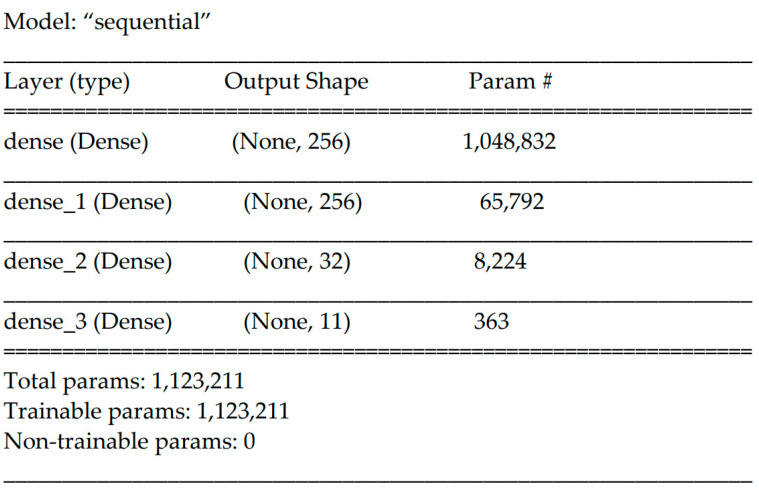
The deep learning model architecture.

We randomly divided the 97 sets of measurement data into a training dataset (75%) and a testing dataset (25%) and fed them into different machine learning algorithm models. Figure 5 shows the different machine learning regression results. The results show that lasso, elastic net, and support vector machine regressor (SVR) are almost completely not working in this case. Gradient boosting, extreme gradient boosting, and decision tree work well for the training data, but not very well for the testing data. Linear regression gives the best results, followed by k-nearest neighbors, partial least squares regression (PLS) and random forest. The deep learning neural network was also used, see Figure 6 for the architecture. It worked fine for the training data, but not very well for the testing data.

### 3.2. Regression—Non-Homogenous Model

**Figure 7:**
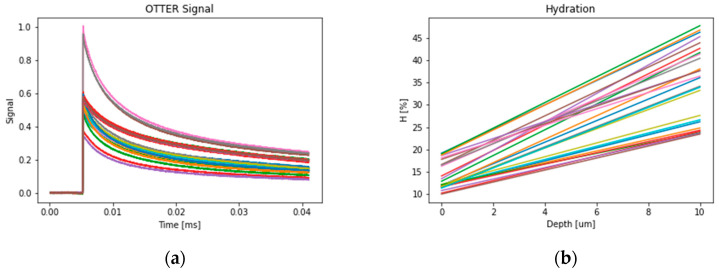
The OTTER skin measurement signals (**a**) and corresponding skin hydration [%] linear distribution depth profiles (**b**). The different colors represent different measurements.

Figure 7 shows the same 97 OTTER skin measurement signals and the corresponding skin hydration depth distributions analyzed by using enhanced segmented least squares (SLS) fitting algorithm, then fitted with Equation (3).

**Figure 8:**
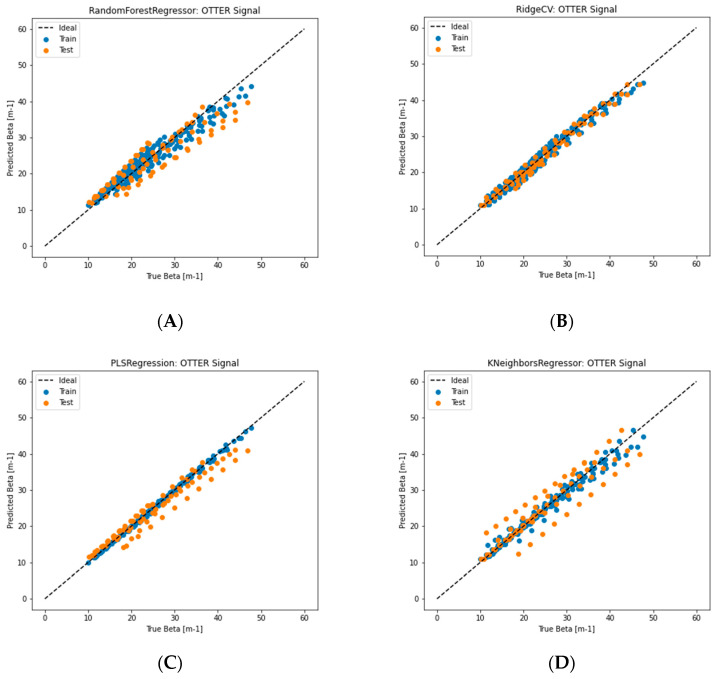

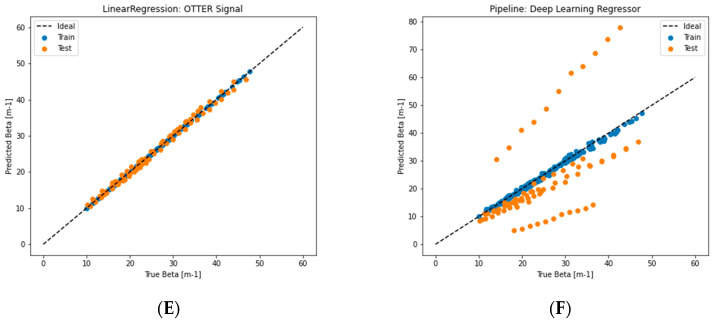
The regression results of different machine learning algorithms models, (**A**) random forest, (**B**) RidgeCV, (**C**) partial least squares (PLS) regression, (**D**) k-nearest neighbours, (**E**) linear regression, (**F**) deep learning neural networks.

Figure 8 shows the different machine learning regression results. As you can see, again, linear regression gives the best result, it works well for both training data and testing data. RidgeCV also gives a very good result, followed by PLS regression and k-nearest neighbor. A deep learning neural network with the same architecture, shown in Figure 6, was also used; again, it does not work very well.

### 3.3. Classification—Real OTTER Data

**Figure 9:**
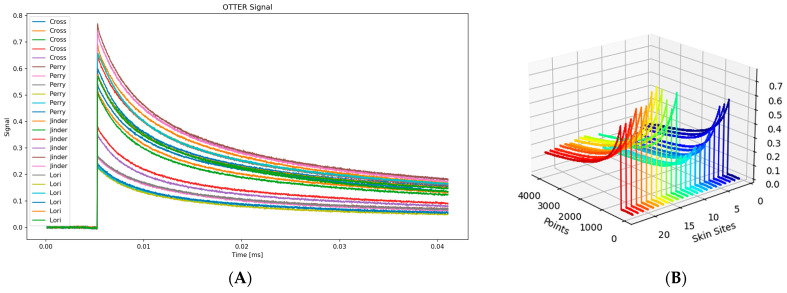
The 20 OTTER signals of four different volunteers on the volar forearm (**A**) and the corresponding 3D presentation (**B**). The different colors represent different measurements.

Figure 9 shows 20 OTTER signals of four different healthy volunteers (male and female, aged 25–55 years old) on the volar forearm, each volunteer has five measurement signals and volunteers are classified as 1, 2, 3, and 4.

The 20 OTTER signals were then randomly divided into a training dataset (75%) and a testing dataset (25%). The training dataset was used to train machine learning models, and trained machine learning models were then tested on the testing dataset. The following are classification results, as shown in Table 1. Accuracy was determined by the percentage of data that a model predicted correctly. Logistic, Naïve-Bayes, AdaBoost, and Gradient Boost obtained the best results, achieving 100% accuracy for training data and 100% accuracy for testing data. The deep learning neural networks model, based on the architecture shown in Figure 7, also performed well and reached 88.2% for training data and 83.3% for testing data.

Linear discriminant analysis (LDA) [62] and principal component analysis (PCA) [63] are two related machine learning algorithms for dimensionality reduction, before later classification. LDA projects the data into a lower dimensioned space to better separate the data into different classes and to reduce computational costs, whilst PCA aims to project the data into new axes (called components), to maximize the variance. LDA first calculates the mean and covariance matrix for each class in the data, then calculates the scatter matrix between classes and that within each class. The goal is to find a projection that can maximize the ratio of the scatter matrix between classes and that within each class. PCA first centers the data around their mean, then finds the eigenvectors and eigenvalues of the covariance matrix, which are then used to project the data onto a lower-dimensional space. The eigenvectors specify the directions of maximum variance, and eigenvalues specify the corresponding amount of variance. The number of principal components represents the amount of variance we want to retain. Typically, we choose a number of principal components that is enough to explain a certain percentage of the total variance in the data.

**Figure 10:**
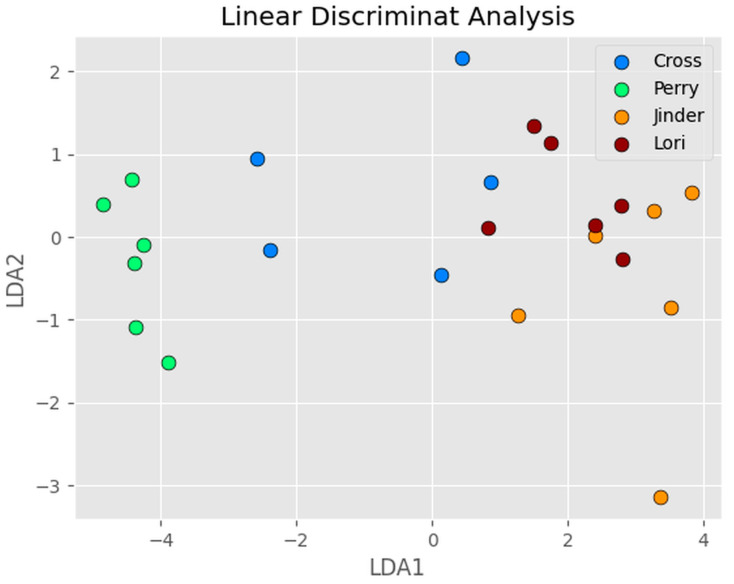
The LDA plot of the first two components of the 20 OTTER signals of four different volunteers on the volar forearm.

Figure 10 shows the LDA plot of the first two components of the 20 OTTER signals of four different volunteers on the volar forearm. The results show that LDA can reasonably separate the OTTER signal from different volunteers effectively, the classification results show that LDA can reach 82.4% accuracy on training data and 83.3% accuracy on testing data.

**Figure 11:**
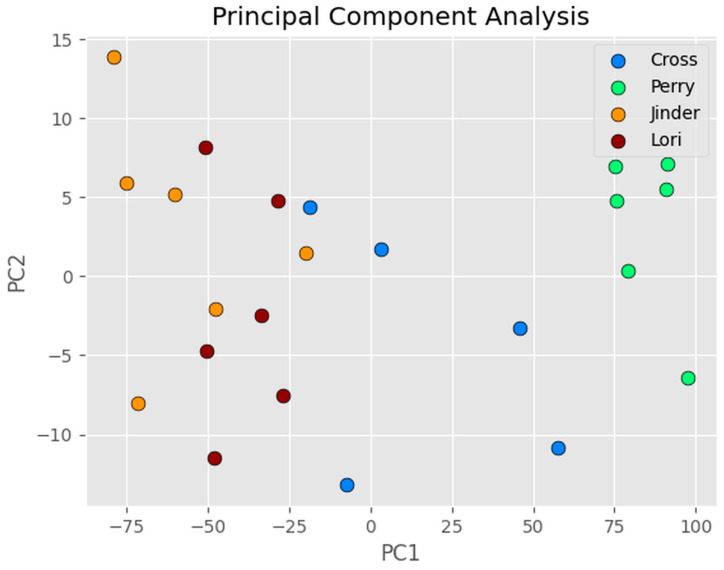
The PCA plot of the first two components of the 20 OTTER signals of four different volunteers on the volar forearm.

Figure 11 shows the PCA plot of the first two components of the 20 OTTER signals of four different volunteers on the volar forearm. The results show that PCA can also reasonably separate the OTTER signal from different volunteers effectively. By applying random forest classifier on PCA results, we can also achieve 100% accuracy when classifying training data and 100% accuracy when classifying testing data.

**Figure 12:**
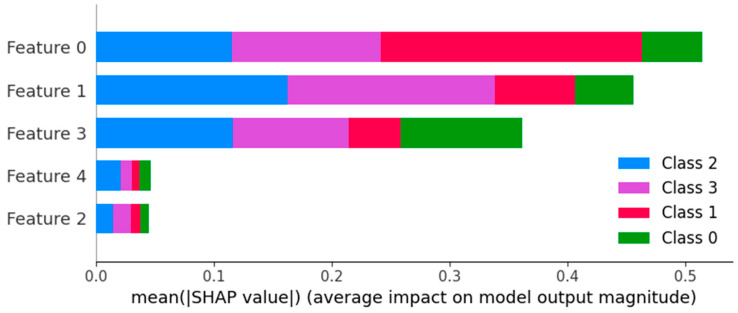
The most important features according to SHAP values.

With SHAP (SHapley Additive exPlanations) [64] values, we can also evaluate the importance of each feature, and how it affects each final prediction. SHAP was originally a game theoretic approach that measures each player’s contribution to the final outcome, and has now been widely using in machine learning to analyze the feature importance. In machine learning, each feature is assigned an important value representing its contribution to the model’s output. By plotting the features according to their importance values, we can understand which are the most important features and which are the least important features. SHAP values can be used to interpret any machine learning model, such as linear regression, decision trees, random forests, gradient boosting models, neural networks, and so on. Figure 12 shows the important features for OTTER data classification. As we are using OTTER signal data values as features, features 0, 1, 2, 3, 4 are the first four data points of the OTTER signal. This means that for classification, the early part of the signal is more important than the later part of the signal.

As for future work, we can further improve the classification accuracy in two ways, fine tuning model hyper parameters [65] and using voting classifier [66].

Most machine learning models have many hyper-parameters and choosing the correct values for the hyper-parameters can have a good impact on the prediction accuracy. Take SVM (support vector machine) for example, which can have the following hyper-parameters: C, the regularization parameter; kernel, the kernel type (‘linear’, ‘poly’, ‘rbf’, ‘sigmoid’, ‘precomputed’, or a callable) to be used in the algorithm; degree, the degree of the polynomial kernel function (‘poly’), ignored by all other kernels, the default degree value is 3; gamma, the kernel coefficient for ‘rbf’, ‘poly’, and ‘sigmoid’. If gamma is ‘auto’, then 1/n_features will be used instead. There are several ways to find the best hyper-parameter values. The simplest one is exhaustive grid search, i.e., search all possible combinations. As you can see, this touch is comprehensive, but could be very time-consuming. An alternative approach is randomized parameter optimization, in which you first randomize the hyper-parameter values, then perform searches for the optimized values.

A voting classifier is a machine learning model that improves classification accuracy by using a collection of models and predicting the results based on the largest majority of votes. It averages each classifier’s results into the voting classifier. There are two different types of voting classifiers: hard voting and soft voting. Hard voting predicts the output with the largest majority of votes. Soft voting averages the probabilities of the classes to determine which one will be the final prediction.

## 4. Conclusions

We have investigated a range of machine learning algorithms for analysing our opto-thermal transient emission radiometry (OTTER) signals. For regression, we have investigated the OTTER signals using both homogenous model and non-homogenous model. For the homogeneous model, the results show that lasso, elastic net, and support vector machine regressor (SVR) did not work at all. Linear regression gave the best results, followed by k-nearest neighbors and random forest. For the non-homogeneous model, linear regression gave the best result, followed by RidgeCV, PLS regressor, and k-nearest neighbors. In both cases, the deep learning neural network model does not work well. For classification, logistic, AdaBoost, and gradient boost gave the best results, achieving 100% accuracy for both training data and testing data. LDA and PCA can effectively separate the OTTER signals from different volunteers. By applying the random forest classifier to PCA results, we can also achieve 100% accuracy in classifying both training data and testing data. With SHAP values, we can understand the importance of the different features. The results show that for classification, the early part of the OTTER signal is more important than the later part of the signal. For future work, we can further improve classification accuracy by fine tuning model hyper parameters and voting classifiers.

The main advantage of machine learning algorithms is that it can learn through training data and, once trained, it can automatically analyze any unseen data, without needing complex mathematical models. The main disadvantage of machine learning algorithms is that many work like a blackbox, so more work is needed for explainable machine learning algorithms.

## Figures and Tables

**Table 1:** The classification accuracy results for Logistic, Naïve Bayes, SVC, Random Forest, Bagging Classifier, Ada Boost Classifier and Gradient Boosting Classifier.

Models	Accuracy (Training) [%]	Accuracy (Test) [%]
Logistic	100.0%	100.0%
Naive Bayes	100.0%	83.3%
SVC	82.4%	83.3%
Random Forest	100.0%	83.3%
Bagging	70.6%	66.7%
Ada Boost	100.0%	100.0%
Gradient Boost	100.0%	100.0%
Deep Learning	88.2%	83.3%
LDA	82.4%	83.3%

## Data Availability

All the data generated during the study are available upon request.
